# Short-Term Effect of Air Pollution on Daily Hospital Visits for Anxiety Disorders in Southern China with Low Pollution Concentrations

**DOI:** 10.3390/toxics13010045

**Published:** 2025-01-09

**Authors:** Xinyuan Zhong, Tingting Guo, Jianghui Zhang, Qiong Wang, Rong Yin, Kunpeng Wu, Qing Zou, Meng Zheng, Brian J. Hall, Andre M. N. Renzaho, Kangning Huang, Wen Chen

**Affiliations:** 1 Department of Medical Statistics, School of Public Health, Sun Yat-sen University, Guangzhou 510080, China; 2 Department of Epidemiology, School of Public Health, Sun Yat-sen University, Guangzhou 510080, China; 3 Center for Global Health Equity, Environmental Studies, New York University Shanghai, Shanghai 200124, China; 4 Translational Health Research Institute, School of Medicine, Western Sydney University, Campbelltown, NSW 2560, Australia; 5 Center for Migrant Health Policy, Sun Yat-Sen University, Guangzhou 510080, China

**Keywords:** air pollution, anxiety disorder, time-series analysis, health effect

## Abstract

The global prevalence and burden of anxiety disorders (ADs) are increasing. However, findings on the acute effects of air pollution on ADs remain inconclusive. We evaluated the effects of short-term exposure to ambient air pollutants, including fine particulate matter (PM_2.5_), inhalable particulate matter (PM_10_), nitrogen dioxide (NO_2_), carbon monoxide (CO), sulfur dioxide (SO_2_), and ozone (O_3_), on daily hospital visits for ADs. A generalized additive model was used to perform a time-series analysis on data from a Southern China city’s medical insurance system between 1 March 2021, and 31 July 2023. Although the daily levels of most pollutants (PM_10_, SO_2_, CO, NO_2_ and O_3_) were consistently below China and WHO’s Ambient Air-Quality Standards, significant associations were observed between daily hospital visits for ADs and all six air pollutants. Each interquartile range increase in concentrations resulted in the largest odds ratios of 1.08 (95% *CI*: 1.01, 1.16) at lag1 for PM_2.5_, 1.19 (95% *CI*: 1.05, 1.34) at lag07 for NO_2_, 1.14 (95% *CI*: 1.05, 1.23) at lag02 for CO, 1.12 (95% *CI*: 1.01, 1.25) at lag07 for PM_10_, 1.06 (95% *CI*: 1.01, 1.12) at lag7 for SO_2_ and 1.08 (95% *CI*: 1.01, 1.15) at lag7 for O_3_, respectively. The effects of NO_2_ and CO remained robust across subgroup analyses and sensitivity analyses. Females and middle-aged individuals showed stronger associations than other subgroups. The findings underscore the necessity for public health efforts to alleviate the impact of air pollution on mental health, even in low-concentration settings.

## 1. Introduction

Anxiety disorders (ADs) are characterized by symptoms of fear, nervousness, and worry, in addition to physical symptoms [1]. According to the latest Global Burden of Disease study, ADs are a leading cause of disability worldwide. Between 2010 and 2021, ADs experienced the largest rise in age-standardized disability-adjusted life years (DALYs) rates, with an increase of 16.7% (14.0–19.8) [2]. As of 2021, there were approximately 359 million people suffering from ADs worldwide, and 53 million were suffering in China alone [3]. The increasing burden of ADs highlights the urgent need to identify risk factors that contribute to this rise, as it represents a pressing public health concern.

There has been an increasing focus on the relationship between ambient air pollution and mental disorders in recent years. Mechanistically, exposure to air pollutants has been demonstrated to negatively impact the nervous system, leading to neuroinflammation and oxidative stress, which may result in neurodevelopmental disorders [4,5,6,7]. Current population-based studies, albeit limited, also support the negative impact of air pollution on ADs. A study conducted in the United Kingdom suggests that long-term exposure to nitrogen dioxide (NO_2_) and fine particulate matter (PM_2.5_) may increase the risk of anxiety [8]. These findings mirror those reported in the United States [9] and Korea [10]. However, studies investigating short-term effects of air pollutants on ADs are limited. Although several researchers have reported that short-term exposure to PM_2.5_, NO_2_, inhalable particulate matter (PM_10_), carbon monoxide (CO), sulfur dioxide (SO_2_), and ozone (O_3_) may increase the risks of hospital admissions or outpatient visits for ADs, the evidence remains inconclusive [11,12,13,14]. For instance, Ji et al. [13] found a significant association between PM_10_ and ADs in Qingdao, China, but these findings could not be replicated by Muhsin and colleagues [14] in Sweden. Nonetheless, it is worth noting that the research regarding the impact of air pollution on specific subtypes of mental disorders, especially ADs, remains limited. And most studies do not use a diagnosis of an AD as the outcome, instead choosing to focus on anxiety symptoms. Furthermore, most studies focused on areas with high air pollution concentrations [12,13]; research in regions with low pollution concentrations is relatively scarce. Additionally, much of the existing literature has focused on particulate matter, without comprehensively analyzing the effects of other gaseous pollutants. This raises important questions regarding the impact of acute exposure to multiple air pollutants on severe anxiety episodes, particularly those resulting in hospital visits.

To address these knowledge gaps, we assessed the short-term effects of ambient air pollution (PM_2.5_, PM_10_, NO_2_, SO_2_, CO and O_3_) on daily hospital visits for ADs in a typical city in southern China where the pollution concentrations are relatively low.

## 2. Materials and Methods

### 2.1. Study Settings and Participants

Qingyuan City is located in Guangdong Province, China, and has a total land area of 19,000 square kilometers. It has a subtropical monsoon climate and, in this area, summer is the longest season of the year. Qingyuan City exhibits a diverse topography, characterized by extensive plains in the southeast and a preponderance of mountains and hills in the north and central regions [15], which underscores Qingyuan’s representativeness within the southern region of China. As of 2023, it had nearly 4 million residents, and 3.92 million people had enrolled in basic medical insurance (coverage rate: 98.5%) [15]. Daily data on hospital visits for ADs from 1 March 2021 to 31 July 2023 were obtained from the basic medical insurance system of Qingyuan City, which collects medical records, including inpatient and outpatient information, for all insurance enrollees in the city. Individuals diagnosed with ADs were included in this study. According to the *International Classification of Diseases*, 10th version (ICD-10), the disease codes were F40 and F41 [16]. The information we collected included patients’ gender, age, visit date, end date, disease diagnoses and hospital’s name and address. Patients who were hospitalized outside Qingyuan City were excluded because their previous exposure to air pollution could not be confirmed. The data used in this study were approved by the Biomedical Research Ethics Review Committee of the School of Public Health, Sun Yat-sen University [No.(2020)029].

### 2.2. Air-Quality Data

Daily 24 h mean concentrations of PM_2.5_, PM_10_, NO_2_, SO_2_, and CO and daily maximum 8 h average concentrations of O_3_ were obtained from the National Air-Quality Monitoring System, consistent with previous studies [17]. Daily mean concentrations of all air pollutants were determined by averaging all valid monitoring measurements [13]. There are four air monitoring stations in Qingyuan City, primarily located in the most densely populated districts and counties, which adequately represent the exposure levels for the majority of the population. Additionally, these stations are positioned away from pollution sources such as roads and factories [18], ensuring that the data collected from these locations accurately reflect the overall air pollution levels in the city [19].

### 2.3. Covariates

To account for potential confounding variables, we obtained daily meteorological data for Qingyuan City from the China Meteorological Administration Land Data Assimilation System (CLDAS version 2.0) within the National Weather Data-Sharing System of China. For every grid in Qingyuan, we extracted the daily average values of surface air pressure, precipitation, wind speed, specific humidity, and air temperature. The following formulas were used to estimate the relative humidity [20]:

(1) $$\mathrm{Saturation}\;\mathrm{vapor}\;\mathrm{pressure}=6.112\times e^{17.67\times \frac{\mathrm{air}\;\mathrm{temperature}}{\mathrm{air}\;\mathrm{temperature}+243.5}}$$

(2) $$Actual\;vapor\;pressure=\frac{\mathrm{specific}\;humidity\times airpressure}{0.378\times \mathrm{specific}\;humdity+0.622}$$

(3) $$Relative\;humidity=\frac{actual\;vapor\;pressure}{saturation\;vapor\;pressure}\times 100$$

In addition, information on the day of the week when the hospital was visited and on public holidays was collected. Furthermore, since the study period coincided with the COVID-19 pandemic, the impact of COVID-19 lockdown was also included in the analysis.

### 2.4. Statistical Analyses

Time-series analysis and a generalized additive model (GAM) were utilized to explore the acute effects of air pollution on daily hospital visits for ADs. This method accommodates the temporal variations in the data and enables the repeated examination of the same population under different exposure conditions. Air pollutant concentration data were matched with daily patient count data for ADs by date. Given that the daily patient counts exhibited a quasi-Poisson distribution, we performed an over-dispersed GAM [21]. GAM can simultaneously assess both linear and nonlinear correlations between environmental factors and health effects while adjusting for various confounding variables [22].

Based on previous research [13,17,21] and the minimum generalized cross-validation (GCV), we chose the following covariates in the main model:

(1) A natural spline function of calendar time with 5 degrees of freedom (df) per year to account for unmeasured time and seasonal trends;
(2) A natural spline functions with 2 df for daily mean temperature and 2 df for daily relative humidity to control weather confounders;
(3) An indicator variable for the visit day of week (DOW) to address potential differences between weekdays and weekends (1 = weekends and 0 = weekdays);
(4) An indicator variable for public holidays to accommodate potential holiday-related variations (1 = holidays and 0 = non-holidays);
(5) An indicator variable for the COVID-19 pandemic to accommodate potential changes related to COVID-19 lockdown measures (1 = before 8 January 2023 and 0 = after 8 January 2023). We chose 8 January 2023 as the truncated time point because, after this date, COVID-19 was treated as a Category B infectious disease in China, which meant that there were no more lockdowns [23].

The GAM is as follows:

(4) $$\log E\left(Y_{t}\right)=\alpha +\beta *Z_{t}+ns\left(time,df\right)+ns(temperature,df)+ns\left(relative\;humidity,df\right)+DOW+Holiday+COVID-19$$

where $E\left(Y_{t}\right)$ refers to the expected number of patients seeking treatment for ADs per day at day *t*; $Z_{t}$ denotes the concentrations of a single air pollutant at day *t*; β indicates the coefficient for $Z_{t}$; df represents degrees of freedom; ns refers to a natural spline function; and α is the intercept. The outcomes were presented as odds ratios (*ORs*) with 95% confidence intervals (*CIs*) for anxiety disorder-related hospital visits, corresponding to each interquartile range (*IQR*) increase in air pollutants per day.

Short-term exposure to pollutants was defined based on the pollutant levels on the day of the hospital visit and up to the preceding 7 days [11,16]. Based on prior findings about the lagged impacts of air pollution on health [24], we constructed both single-day lag models (lag0–lag7) and moving average lag models (lag01–lag07) to assess cumulative effects. In the single-day lag models, lag0 indicates the current day’s concentration, and lag7 indicates the concentration seven days prior. In the moving average lag models, lag01 represents the average pollutant concentration over the current and previous day, and lag07 represents the average concentration of the current day and the previous seven days. By including a natural spline function with three degrees of freedom for each pollutant in the aforementioned GAM, we were able to plot exposure–response (E–R) relationship curves [25,26].

Additionally, we performed subgroup analyses to investigate potential modifications by age (<45, 45–64, ≥65) and gender (female, male). Moreover, $(Q_{1}-Q_{2})\pm 1.96\sqrt{SE_{1}^{2}+SE_{2}^{2}}$ was used to examine the differences between each two subgroups, where $Q_{1}$, $Q_{2}$ represented effect estimates of two subgroups and $SE_{1}$ and $SE_{2}$ were their corresponding standard errors [27,28].

We performed three sensitivity studies to confirm the model’s stability. First, we used varying degrees of freedom (df = 3 to 7) to correct for seasonal and long-term trends. Secondly, we added co-pollutants with a Spearman’s correlation coefficient less than 0.7 to the main model in order to fit two-pollutant models [29]. For each air pollutant, the lag period with the largest impact was chosen. Thirdly, we analyzed data collected only before 8 January 2023.

We used R software version 4.3.2 to complete the analysis, and the *p* value < 0.05 was considered statistically significant.

## 3. Results

### 3.1. General Characteristics

A total of 2784 hospital visits for ADs, including inpatients and outpatients, were observed between 1 March 2021 and 31 July 2023. An average of 3.15 patients were admitted every day. The mean age of the patients was 52.54 (standard deviation = 14.94 years old). (Table 1). A time-series plot of the number of daily hospital visits for ADs is shown in Figure S1 of the Supplementary Materials.

As shown in Table 2, the daily concentrations of six air pollutants in Qingyuan City were all below China’s Ambient Air-Quality Standards. Additionally, except for PM_2.5_, the others were also below the World Health Organization 2021 Global Air-Quality Guidelines. At the same time, the daily average temperature and relative humidity were 21.03 °C and 78.17%. Concentrations of air pollutants, except for O_3_, were higher in winter and lower in summer (see Supplementary Figure S2).

**Table 2:** Descriptive statistics for daily concentrations of air pollutants, and meteorological factors.

	Mean	SD	Min	P_25_	P_50_	P_75_	Max	IQR	CHINA	WHO
Air pollutant concentration										
PM_2.5_ (μg/m^3^)	21.67	12.18	0.00	13.00	19.00	29.00	76.00	16.00	75	15
PM_10_ (μg/m^3^)	36.04	20.27	0.00	22.00	32.00	47.00	145.00	25.00	150	45
SO_2_ (μg/m^3^)	6.81	1.81	3.00	6.00	7.00	8.00	14.00	2.00	150	40
NO_2_ (μg/m^3^)	19.32	8.80	4.00	13.00	18.00	23.50	57.00	10.50	80	25
CO (mg/m^3^)	0.70	0.19	0.20	0.60	0.70	0.80	1.50	0.20	4	4
O_3_ (μg/m^3^)	96.48	45.42	3.00	63.00	96.48	128.00	261.00	65.00	160	100
Meteorological measure										
Mean temperature (℃)	21.03	6.77	3.79	15.81	22.25	26.92	30.83	11.11	-	-
Relative humidity (%)	78.17	11.43	32.45	72.30	79.29	87.28	95.08	14.98	-	-
Surface air pressure (hpa)	973.1	5.89	958.4	968.6	972.6	977.5	989.0	8.93	-	-
Precipitation (mm)	5.65	10.71	0.00	0.01	0.82	6.27	83.23	6.26	-	-
Wind speed (m/s)	0.94	0.32	0.50	0.71	0.85	1.05	2.76	0.34	-	-

Abbreviations: PM_2.5_—fine particulate matter; PM_10_—inhalable particulate matter; NO_2_—nitrogen dioxide; CO—carbon monoxide; SO_2_—sulfur dioxide; O_3_—ozone; P_25_, P_50_, P_75_—the 25th, 50th, and 75th percentile; Min—minimum; Max—maximum; SD—standard deviation; IQR—interquartile range; CHINA—China’s Ambient Air-Quality Standards [30]; WHO—the World Health Organization 2021 Global Air-Quality Guidelines [31].

The Spearman correlation coefficients among all air pollutants were significant, except for those of CO and SO_2_. A strong correlation (*r* = 0.95) was observed between PM_2.5_ and PM_10_ (see Supplementary Table S1).

### 3.2. Single-Pollutant Model Results for Hospital Visits for Anxiety Disorders

**Figure 1:**
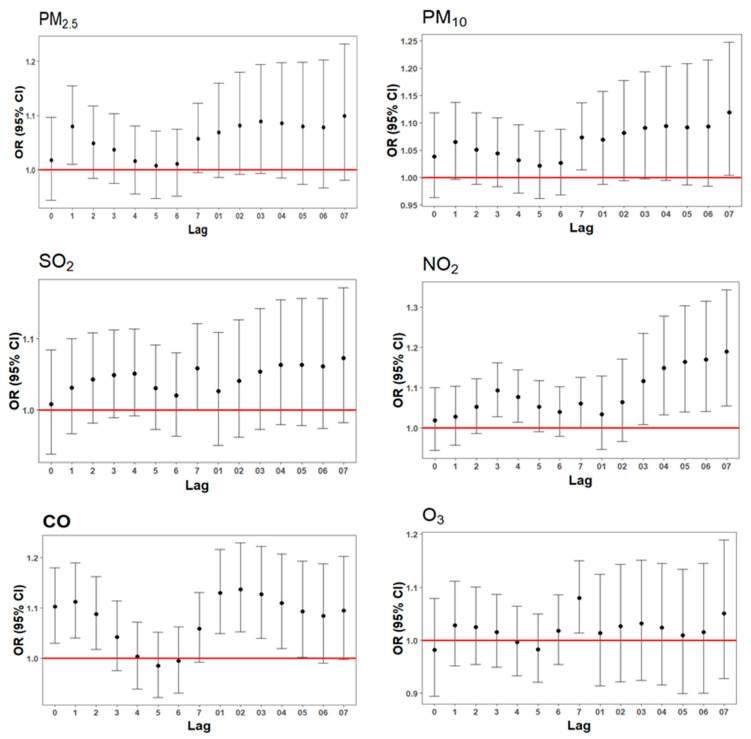
Impact of PM_2.5_, PM_10_, NO_2_, CO, SO_2_, and O_3_ on daily anxiety-related hospital visits at different lag days (red line means an *OR* = 1).

Overall, short-term exposure to PM_2.5_, PM_10_, SO_2_, NO_2_, CO, and O_3_ was positively correlated with the daily hospital visits for ADs (Figure 1). PM_2.5_ was significant at lag1 day, with each *IQR* increase corresponding to an *OR* of 1.08 (95% *CI*: 1.01, 1.16). PM_10_ was significant at lag7 day and lag07 day, with the strongest effect observed at lag07 day (*OR* = 1.12, 95% *CI*: 1.00, 1.25). SO_2_ was significant at lag7 day (*OR* = 1.06, 95% *CI*: 1.00, 1.12). NO_2_ showed statistical significance at lag3–4 day and lag03–07 day, with the strongest effect at lag07 day (*OR* = 1.19, 95% *CI*: 1.05, 1.34). CO was significant at lag0–2 day and lag01–05 day, with the strongest effect observed at lag02 day (*OR* = 1.14, 95% *CI*: 1.05, 1.23). O_3_ was significant at lag7 day (*OR* = 1.08, 95% *CI*: 1.01, 1.15) (see Supplementary Table S2).

Exposure–response curves for PM_10_, NO_2_, SO_2_ and O_3_ were linear, with no significant thresholds identified. The curves of PM_2.5_ and CO exhibited steep slopes at low concentrations and became flat at higher concentrations (see Supplementary Figure S3).

### 3.3. Subgroup Analyses

#### 3.3.1. Effect by Gender

**Figure 2:**
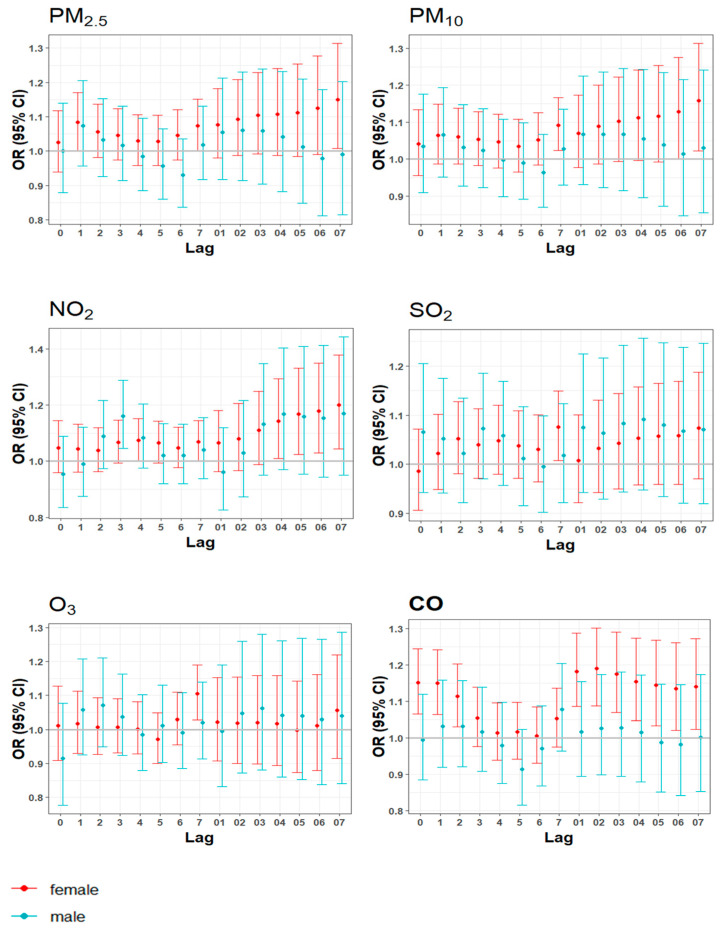
Gender-specific analysis for daily anxiety-related hospital visits and air pollutants.

As shown in Figure 2, the results for females were similar to those for the whole population. PM_2.5_ was significant at lag1, lag7, and lag07 day. PM_10_ was statistically significant at lag7 and lag07 day. NO_2_ was statistically significant at lag4 and lag04–07 day. SO_2_ and O_3_ were significant at lag7 day. CO was significant at lag0–2 and lag01–07 day. On the contrary, the correlations in males were insignificant. NO_2_ was statistically significant only at lag3 day, with an *OR* of 1.16 (95% *CI*: 1.04,1.29) (see Supplementary Table S3).

Overall, females exhibited greater associations than males, although gender differences were significant only at lag0 day for CO (see Supplementary Table S4).

#### 3.3.2. Effect by Age

**Figure 3:**
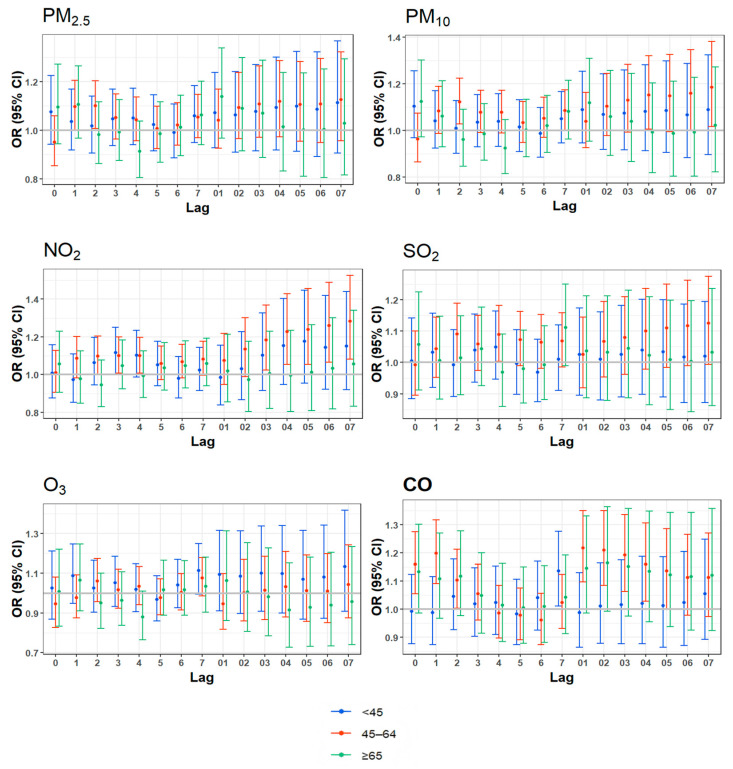
Age-specific analysis for daily anxiety-related hospital visits and air pollutants.

As shown in Figure 3, in the younger group (<45 years old), only CO showed a positive correlation at lag7 day with an *OR* of 1.14 (95% *CI*: 1.01, 1.28). In the middle-aged group (45–64 years old), significant correlations were observed at lag2 day for PM_2.5_; at lag2, lag7, lag04, and lag07 day for PM_10_; at lag2 and lag4 day for SO_2_; at lag2–4 and lag03–07 day for NO_2_; and at lag0–2 and lag01–05 day for CO, respectively. In the older group (≥65 years old), none of the air pollutants showed statistical significance (see Supplementary Table S5).

Overall, the middle-aged group showed greater associations than other age groups. However, the differences between the younger group and the older group were not significant. The effects of CO and SO_2_ on ADs showed significant differences between the younger group and the middle-aged group, with stronger associations observed in the middle-aged group. The effect of PM_10_ on ADs differed significantly between the middle-aged group and the older group, and the association was slightly stronger in the middle-aged group (see Supplementary Tables S6–S8).

### 3.4. Sensitivity Analyses

The effect estimates of CO and NO_2_ were robust under different *df* values of calendar time, while other estimated effects changed slightly (see Supplementary Table S9). In addition, the two-pollutant models indicated that the associations for CO and NO_2_ remained robust after all other pollutants were added. PM_2.5_ became nonsignificant only after the addition of CO. PM_10_ remained significant only after the addition of O_3_. SO_2_ and O_3_ maintained significance only after the addition of CO (see Supplementary Table S10). Restricting the analysis to data obtained before 8 January 2023 ensured that the models produced similar results (see Supplementary Table S11).

## 4. Discussion

This study explored the association between short-term air pollution exposure and hospital visits for ADs in a city in Southern China, where the daily concentrations of most pollutants (PM_10_, SO_2_, NO_2_, CO, and O_3_) were consistently below China and the WHO’s Ambient Air-Quality Standards. Our results suggested that even at relatively low pollution levels, short-term exposure to these pollutants, particularly NO_2_ and CO, increased the risk of hospital visits for ADs. In subgroup analyses, females and individuals aged 45–64 years were more susceptible to air pollutants, although most differences between subgroups were not significant. Our study addressed the inadequate information regarding acute effects of air pollutants on the hospital visits for ADs in areas with low pollution concentrations.

Previous studies have often analyzed the effects of air pollution in regions with high pollutant concentrations, particularly in Northern China or industrialized countries where pollution levels exceed recommended thresholds. For example, studies conducted in cities like Shenyang and Qingdao have primarily focused on higher pollution levels, which may yield stronger associations with anxiety-related outcomes [12,13]. In contrast, our study was conducted in a region with low ambient pollution, providing insight into the effects of air pollutants even when concentrations are below health guidelines. This adds an important dimension to the growing body of literature by showing that the health risks posed by air pollution can persist at lower levels, which is critical for shaping public health interventions in regions with improving air quality. In addition, the exposure–response curves for most pollutants are linear, indicating no clear threshold. This suggests that it is crucial to reduce pollution sources, rather than merely lowering pollutant concentrations below a certain level. Moreover, although the ozone concentration in our study was below the WHO’s guideline, at 96.48 µg/m^3^, this level remains relatively high. In recent years, ozone pollution has become an increasingly prominent issue. Jin et al. [32] and Xu et al. [33] have also found that ozone is associated with an increased risk of ADs, though current evidence remains insufficient. Ozone, as a potent oxidizing agent, can cause oxidative damage and activate pro-inflammatory molecules [34]. Additionally, ozone may increase the release of stress hormones and glucocorticoids by activating the hypothalamic–pituitary–adrenal (HPA) axis [35]. Both of these factors play critical roles in the pathophysiology of mental disorders. Therefore, further research is needed to explore the impact of ozone on ADs and other mental health issues. Overall, our observed associations may provide additional insights into the effects of ambient pollutants in low-concentration sites.

Unlike most longitudinal design studies that examined long-term air pollution exposure [8,9,10,36,37], we focused on short-term effect and anxiety disorder-related hospital visits. Existing evidence suggests that acute increases in ambient air pollution levels are associated with the occurrence of acute health events. For instance, Muhsin et al. found that acute exposure to air pollution resulted in a significant deterioration in mental health [14]. Acute increases in ambient air pollution may exacerbate symptoms in individuals who are already experiencing anxiety, potentially leading to an increased likelihood of hospital visits. Some evidence is available to support the effects of short-term exposures, particularly with regard to depression [17], but fewer studies have focused on anxiety and the findings are inconsistent [11,13,16,38]. In addition, some studies have shown that the short-term effects of air pollution on ADs are delayed. For example, Yue et al. reported a positive correlation between PM_2.5_, PM_10_, and hospital admissions for ADs at lag2–lag6, as well as at lag01 and lag05 [16]. Ma et al. observed that SO_2_ was significantly associated with an increased risk of daily hospital admissions for ADs at lag0, lag1, lag4–7, and lag01–07 [11]. Similarly, our study also identified lagged effects of air pollution on hospital visits for ADs. One possible explanation is that patients exhibiting symptoms often delay seeking medical attention, opting to wait and see whether their condition improves spontaneously [39]. Moreover, most hospital visits for ADs are non-urgent. In China, it is common to schedule appointments before visiting the hospital, typically resulting in a delay of several days. The biological mechanisms underlying this lagged association require further investigation.

Our study showed that PM_2.5_, PM_10_, NO_2_, CO, SO_2_, and O_3_ have adverse effects on ADs. A multicity study conducted in China supported our findings, although it concentrated on only particulate matter [16]. Another study in Qingdao also found that short-term exposure to PM_2.5_, PM_10_, NO_2_ and CO increased the risk of daily anxiety hospitalizations [13]. However, another multi-city study in China identified significant associations between NO_2_, SO_2_, and anxiety admissions, while no notable correlations were found with PM_2.5_, PM_10_, CO, and O_3_ [11]. A study in Shenyang found that only SO_2_ and CO had significant effects on outpatients visits related to anxiety [38]. This discrepancy could be attributed to several factors. First, variations may arise due to differences in study locations and populations. Hu et al. reported that the infiltration and exposure levels of air pollutants were greater in southern China compared to northern China [40]. Second, the toxicity of air pollutants may primarily depend on their chemical composition. According to prior research, the health impacts of particle compositions may outweigh the negative effects associated with their concentrations [41]. Therefore, further research is necessary to investigate the impact of particle compositions on ADs.

Notably, our results found that gaseous pollutants (NO_2_ and CO) remained robust in both subgroup analysis and sensitivity analysis. Therefore, CO and NO_2_ may be key air pollutants that contribute to an increased risk of ADs. CO is primarily generated by anthropogenic activities, including tailpipe emissions. NO_2_, which is mainly produced via fossil fuel combustion, contributes to the formation of environmentally harmful O_3_. Both CO and NO_2_, along with other air pollutants, have toxic effects on the brain, leading to neuroinflammation, neurodegeneration, and cerebral vascular damage [42]. An experimental study found that inhalation of NO_2_ altered the expression of related genes, inducing anxiety-like mental disorders in adult mice [43]. Additionally, CO and NO_2_ are pro-oxidants, potentially increasing oxidative stress levels upon prolonged exposure, which may contribute to heightened anxiety and depression-like behaviors [44].

In subgroup analyses, for the gender-stratified result, we found that the number of females with ADs was twice that of males, aligning with previous studies [12,13]. Additionally, females exhibited higher *ORs* for PM_2.5_ and CO exposure than males, with this gender difference being statistically significant for CO. This discrepancy may be attributed to variations in hormone levels [45], neurobiological processes [46], and variation in lung structure and reactivity, which affect airway resistance and exposure differences [47]. Kim et al. reported that the pulmonary deposition of particulate matter was significantly higher in females than in males [48], which may contribute to the increased sensitivity observed in females. Additionally, indoor burning of fossil fuels for cooking significantly elevates PM_2.5_ and CO concentrations in kitchens [49], and females in China typically engage in more cooking than males, leading to higher exposure levels. Furthermore, behavioral studies have shown that males are generally less willing than females to seek help for mental health-related issues [50]. In terms of age-stratified results, the 45–64 years age group had a higher rate of hospital visits for ADs than other age groups, and there was a stronger correlation between ADs and air pollutants in this group. Our results are partially supported by previous studies. Cao et al. demonstrated a significant association between PM_2.5_ and ADs in individuals aged over 45 [51]. Additionally, Ma et al. documented a stronger correlation between SO_2_ and anxiety-related hospitalizations in patients under 65 years old [11]. However, two studies reported no significant variations in different age groups [12,16]. Further research is necessary to incorporate additional influencing factors, including educational attainment, economic status, and the presence of chronic diseases. Overall, the subgroup results could aid in identifying vulnerable populations and support targeted disease prevention efforts related to environmental exposures.

Our study has several limitations. Firstly, we utilized averaged exposure data from monitoring stations within the city, which may lead to inaccuracies in exposure-level assessments. For example, monitoring stations are primarily located in densely populated districts and counties, where pollution levels are commonly higher than remote areas, potentially leading to exposure being overestimated for some individuals. Secondly, in this study, we were unable to precisely match exposure levels based on residential locations, as the individual’s home address was not provided in the authorized medical insurance data. Thirdly, the number of hospital visits for ADs is limited. Nevertheless, a quasi-Poisson-distributed GAM can accommodate small counts [52], thereby mitigating the impact of sample size to some extent. Additionally, several potential confounders, such as comorbidities, were not included in this study, and these should be investigated further in the future. However, this study also has some advantages. Firstly, most prior research has been limited to large hospitals or hospitals specializing in psychiatry, which restricts their representativeness in the general population. Our study used basic medical insurance data covering 3.92 million people, with a coverage rate of 98.5%, providing better representativeness. In addition, the anxiety-disorder patients we included were diagnosed by doctors, avoiding the inaccuracies associated with self-reported questionnaires.

## 5. Conclusions

Our study indicates that short-term exposure to ambient air pollutants, particularly gaseous pollutants (NO_2_ and CO), is associated with a heightened risk of hospital visits for ADs in a Southern China city with low pollution concentrations. The effects appear stronger in females and in adults aged 45–64. These findings further confirm that air pollution is a risk factor for ADs. Given the increasing burden of ADs and the fact that air pollution is modifiable, there is a need to prevent ADs by reducing the levels of these pollutants.

## Figures and Tables

**Table 1:** Descriptive statistics for daily anxiety disorders visits during the study period.

	N (%)	Mean	SD
Overall	2784 (100)	3.15	2.70
Gender			
Male	823 (29.56)	0.93	1.12
Female	1961 (70.44)	2.22	2.07
Age, years			
<45	830 (29.81)	0.94	1.16
45–64	1345 (48.31)	1.52	1.71
≥65	609 (21.88)	0.69	0.94
Year of hospital visits			
2021	1162 (41.74)	3.80	2.46
2022	1391 (49.96)	3.81	2.93
2023	231 (8.3)	1.09	1.12
Levels of Hospital			
Tertiary hospitals	540 (19.40)	0.61	0.86
Secondary hospitals	1934 69.47)	2.19	2.20
Primary hospitals	310 (11.13)	0.31	0.67
Type of hospital visit			
Outpatient visits	2195 (78.84)	2.49	2.62
Hospitalization	589 (21.16)	0.67	0.89

Abbreviations: SD, standard deviation; primary hospitals refer to community-level healthcare facilities, which primarily focus on basic healthcare services; secondary hospitals are regional healthcare facilities serving multiple communities; and tertiary hospitals are comprehensive medical, teaching, and research centers with advanced technical capabilities.

## Data Availability

The exposure data are available from https://www.aqistudy.cn/ (accessed on 20 March 2024). Patient data are available upon request to the corresponding author.

## Supplementary Materials

The following supporting information can be downloaded at https://www.mdpi.com/article/10.3390/toxics13010045/s1, Figure S1: A time-series plot of the number of daily anxiety visits; Figure S2: Time-series plots of air pollutants during the study period; Figure S3: The exposure–response curves of air pollutants with daily visits for anxiety disorders. The lines represent the point estimates and the shadings indicate corresponding 95% *CI*s. Table S1: Spearman’s correlations between air pollutants and meteorological factors; Table S2: The results (*OR* with 95% *CI*) of daily anxiety visits associated with *IQR* increase in PM_2.5_, PM_10_, CO, SO_2_, NO_2_ and O_3_ for different lag structures; Table S3: The results (*OR* with 95% *CI*) of daily anxiety visits associated with *IQR* increase in PM_2.5_, PM_10_, CO, SO_2_, NO_2_ and O_3_ stratified by sex; Table S4: Comparison of the effects of air pollutants on daily anxiety visits in different gender groups; Table S5: The results (*OR* with 95% *CI*) of daily anxiety visits associated with *IQR* increase in PM_2.5_, PM_10_, CO, SO_2_, NO_2_ and O_3_ stratified by age; Table S6: Comparison of the effects of air pollutants on daily anxiety visits between young-aged group and middle-aged group; Table S7: Comparison of the effects of air pollutants on daily anxiety visits between young-aged group and old-aged group; Table S8: Comparison of the effects of air pollutants on daily anxiety visits between middle-aged group and old-aged group; Table S9: The results (*OR* with 95% *CI*) of daily anxiety visits associated with *IQR* increase in PM_2.5_, PM_10_, SO_2_, NO_2_ CO and O_3_ for different degrees of freedom; Table S10: The results (*OR* with 95% *CI*) of daily anxiety visits associated with *IQR* increase in PM_2.5_, PM_10_, CO, SO_2_, NO_2_ and O_3_ in two-pollutant models; Table S11: The results (*OR* with 95% *CI*) of daily anxiety visits associated with *IQR* increase in PM_2.5_, PM_10_, CO, SO_2_, NO_2_ and O_3_ for different lag structures, restricting analysis before 8 January 2023.
