# Supplementary material for: Short-Term Effect of Air Pollution on Daily Hospital Visits for Anxiety Disorders in Southern China with Low Pollution Concentrations
Source: Toxics. 2025 Jan 9;13(1):45. doi: 10.3390/toxics13010045 (PMC11768768; doi:10.3390/toxics13010045)
Supplement: Supplementary file 1 [file toxics-13-00045-s001.zip › toxics-3392420-supplementary.pdf]

Figure S1 A time series plot of the number of daily anxiety visits 3
Figure S2 Time series plots of air pollutants during the study period 4
Figure S3 The exposure-response curves of air pollutants with daily visits for anxiety disorders. The lines represent the point estimates and the shadings indicate corresponding 95% *CI*s. 5
Table S1 Spearman's correlations between air pollutants and meteorological factors 6
Table S2 The results (OR with 95% CI) of daily anxiety visits associated with IQR increase in $PM_{2.5}$, PM_{10}, CO, SO_2, NO_2 and O_3 for different lag structures 7
Table S3 The results (OR with 95% *CI*) of daily anxiety visits associated with IQR increase in $PM_{2.5}$, PM_{10}, CO, SO_2, NO_2 and O_3 stratified by sex. 8
Table S4 Comparison of the effects of air pollutants on daily anxiety visits in different gender groups 10
Table S5 The results (OR with 95% *CI*) of daily anxiety visits associated with IQR increase in $PM_{2.5}$, PM_{10}, CO, SO_2, NO_2 and O_3 stratified by age 11
Table S6 Comparison of the effects of air pollutants on daily anxiety visits between young-aged group and middle-aged group 14
Table S7 Comparison of the effects of air pollutants on daily anxiety visits between young-aged group and old-aged group.. 15
Table S8 Comparison of the effects of air pollutants on daily anxiety visits between middle-aged group and old-aged group... 16
Table S9 The results (OR with 95% *CI*) of daily anxiety visits associated with IQR

increase in $PM_{2.5}$, PM_{10}, SO_2, NO_2 CO and O_3 for different degrees of freedom 17

Table S10 The results (OR with 95% *CI*) of daily anxiety visits associated with IQR increase in $PM_{2.5}$, PM_{10}, CO, SO_2, NO_2 and O_3 in two-pollutant models.. 17

Table S11 The results (OR with 95% CI) of daily anxiety visits associated with IQR increase in $PM_{2.5}$, PM_{10}, CO, SO_2, NO_2 and O_3 for different lag structures, restricting analysis before January 8, 2023. 19

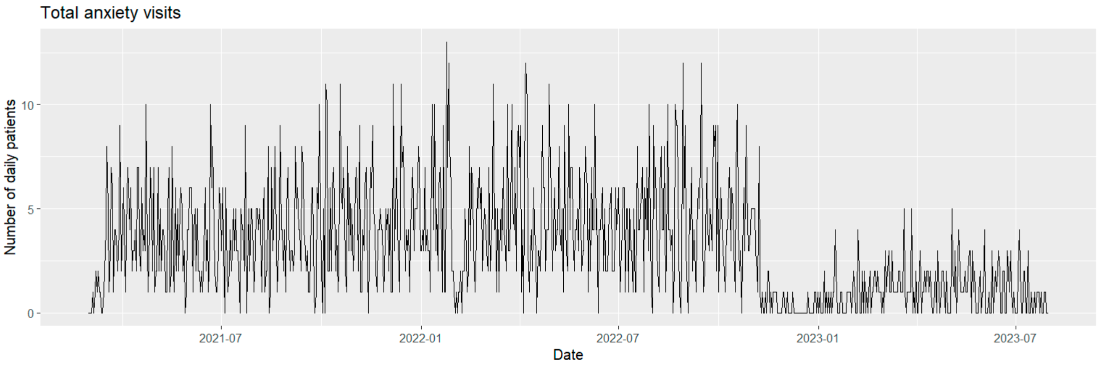

Figure S1 A time series plot of the number of daily anxiety visits

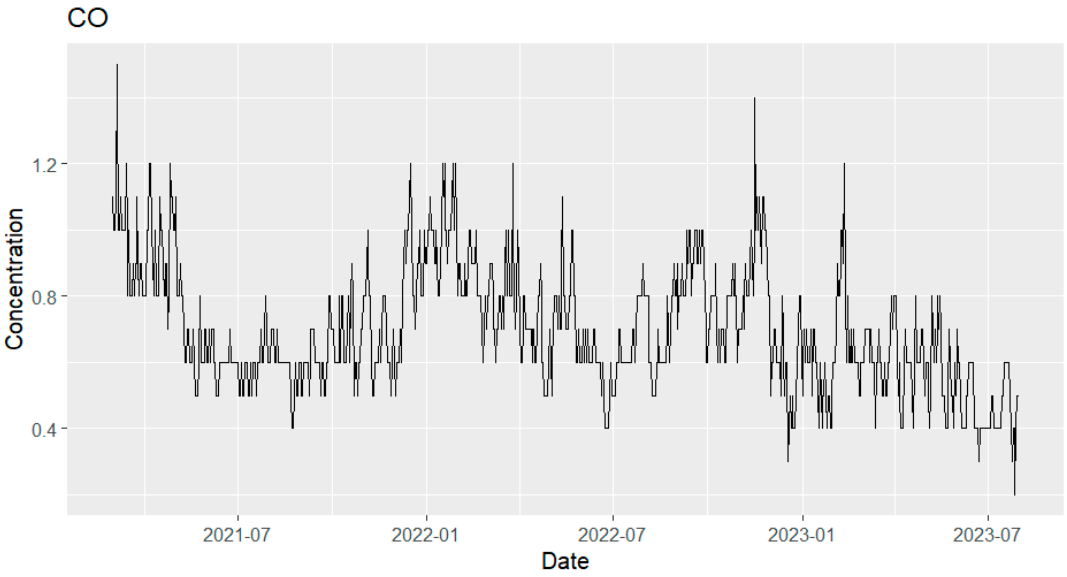

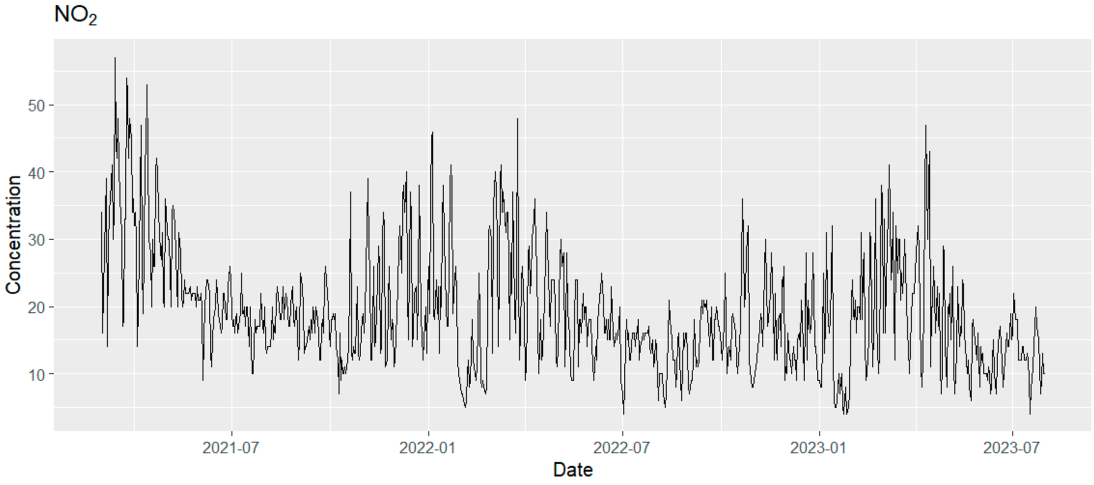

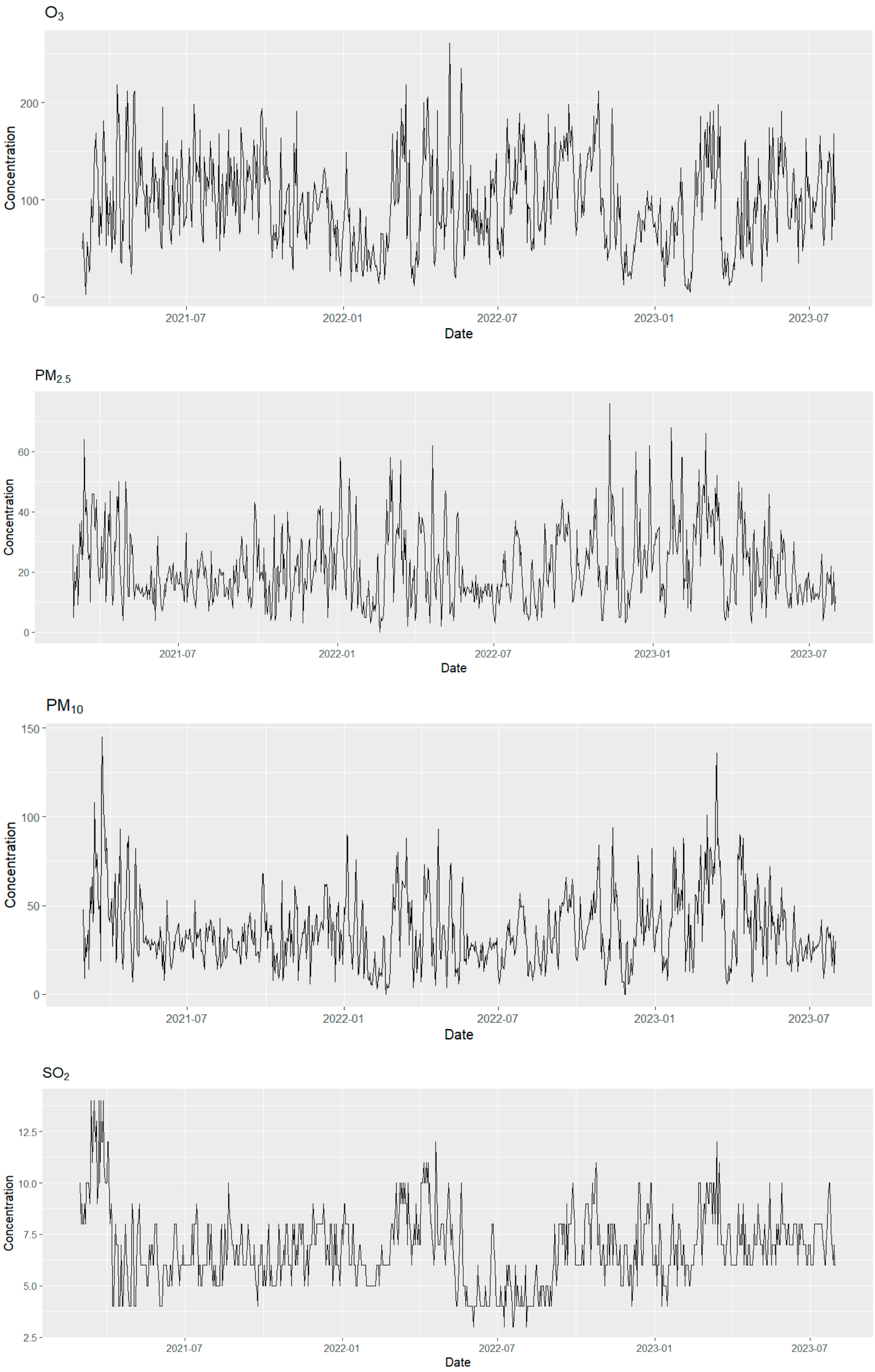

Figure S2 Time series plots of air pollutants during the study period

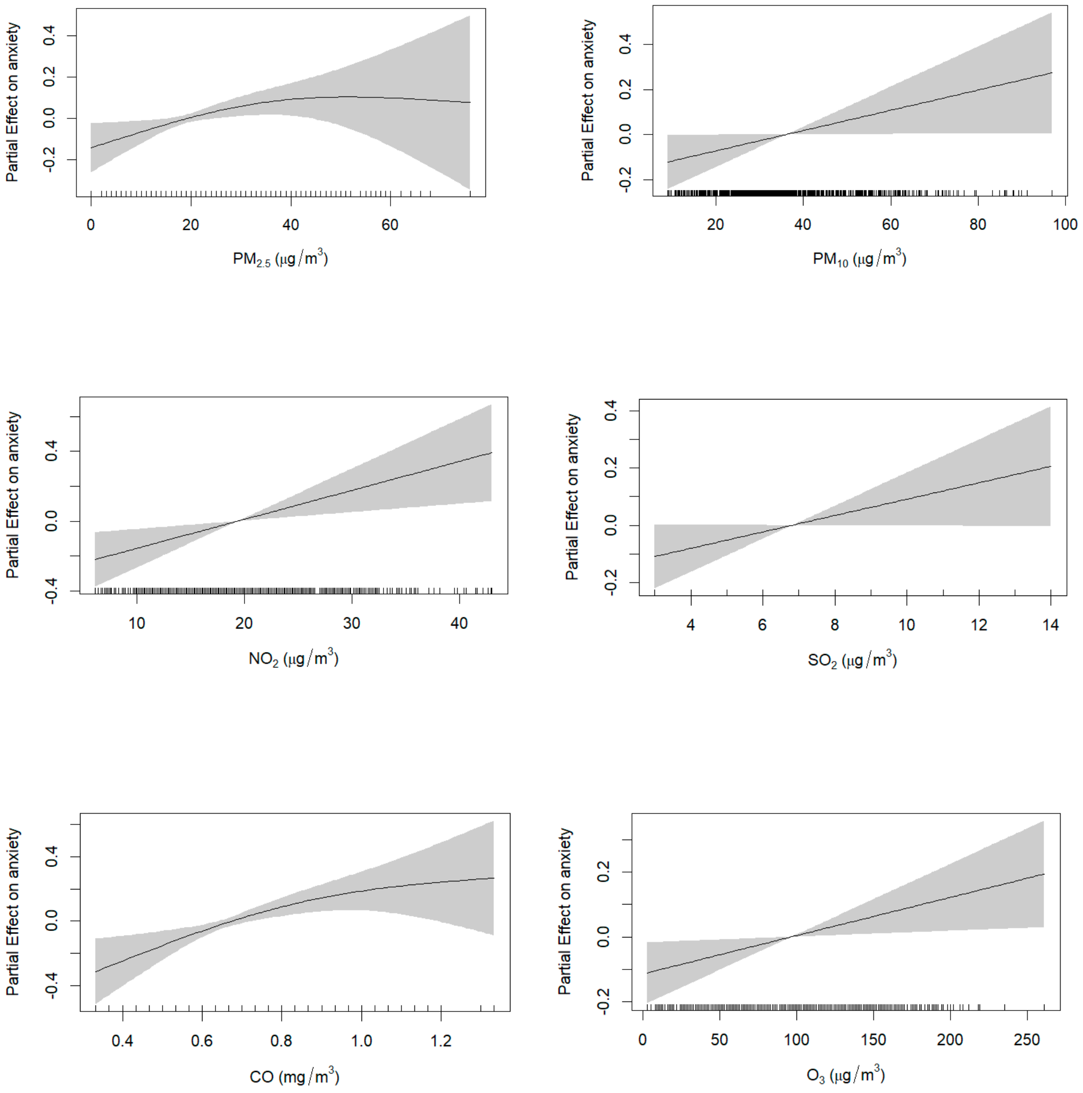

Figure S3 The exposure-response curves of air pollutants with daily visits for anxiety disorders. The lines represent the point estimates and the shadings indicate corresponding 95% *CI*s.

Table S1 Spearman's correlations between air pollutants and meteorological factors

	$PM_{2.5}$	PM_{10}	SO_2	NO_2	CO	O_3	WIN	TMP	RH	PRS	PRE
$PM_{2.5}$	1.00										
PM_{10}	**0.95***	1.00									
SO_2	0.54*	0.60*	1.00								
NO_2	0.54*	0.57*	0.40*	1.00							
CO	0.29*	0.18*	0.03	0.36*	1.00						
O_3	0.49*	0.57*	0.36*	0.22*	-0.08*	1.00					
WIN	-0.13*	-0.12*	0.06	-0.18*	0.05	-0.28*	1.00				
TMP	-0.05	0.03	-0.11*	-0.04	-0.33*	0.51*	-0.43*	1.00			
RH	-0.39*	-0.45*	-0.43*	0.04	0.12*	-0.57*	-0.08*	0.00	1.00		
PRS	0.17*	0.11*	0.22*	0.03	0.28*	-0.21*	0.30*	**-0.82***	-0.32*	1.00	
PRE	-0.39*	-0.41*	-0.39*	-0.08*	-0.08*	-0.38*	-0.10*	0.19*	**0.81***	-0.44*	1.00

*$P < 0.05$; correlation coefficients greater than 0.7 are highlighted in bold;

WIN: wind speed, TMP: temperature, RH: relative humidity, PRS: surface air pressure, PRE: precipitation.

Table S2 The results (OR with 95% CI) of daily anxiety visits associated with IQR increase in $PM_{2.5}$, PM_{10}, CO, SO_2, NO_2 and O_3 for different lag structures

Lag days	$PM_{2.5}$	PM_{10}	CO	NO_2	SO_2	O_3
0	1.02(0.94, 1.10)	1.04(0.96, 1.12)	**1.10(1.03, 1.18) ***	1.02(0.94, 1.10)	1.01(0.94, 1.08)	0.98(0.89, 1.08)
1	**1.08(1.01, 1.16)**	1.06(1.00, 1.14)	**1.11(1.04, 1.19) ***	1.03(0.96, 1.10)	1.03(0.97, 1.10)	1.03(0.95, 1.11)
2	1.05(0.98, 1.12)	1.05(0.99, 1.12)	**1.09(1.02, 1.16)**	1.05(0.99, 1.12)	1.04(0.98, 1.11)	1.02(0.95, 1.10)
3	1.04(0.97, 1.10)	1.04(0.98, 1.11)	1.04(0.98, 1.11)	**1.09(1.03, 1.16)**	1.05(0.99, 1.11)	1.02(0.95, 1.09)
4	1.02(0.96, 1.08)	1.03(0.97, 1.10)	1.00(0.94, 1.07)	**1.08(1.01, 1.14)**	1.05(0.99, 1.11)	1.00(0.93, 1.06)
5	1.01(0.95, 1.07)	1.02(0.96, 1.08)	0.98(0.92, 1.05)	1.05(0.99, 1.12)	1.03(0.97, 1.09)	0.98(0.92, 1.05)
6	1.01(0.95, 1.07)	1.03(0.97, 1.09)	0.99(0.93, 1.06)	1.04(0.98, 1.10)	1.02(0.96, 1.08)	1.02(0.95, 1.09)
7	1.06(1.00, 1.12)	**1.07(1.01, 1.14)**	1.06(0.99, 1.13)	1.06(1.00, 1.13)	**1.06(1.00, 1.12)**	**1.08(1.01, 1.15)**
01	1.07(0.99, 1.16)	1.07(0.99, 1.16)	**1.13(1.05, 1.22) ***	1.03(0.95, 1.13)	1.03(0.95, 1.11)	1.01(0.91, 1.12)
02	1.08(0.99, 1.18)	1.08(0.99, 1.18)	**1.14(1.05, 1.23) ***	1.06(0.97, 1.17)	1.04(0.96, 1.13)	1.03(0.92, 1.14)
03	1.09(0.99, 1.19)	1.09(1.00, 1.19)	**1.13(1.04, 1.22) ***	**1.12(1.01, 1.24)**	1.05(0.97, 1.14)	1.03(0.92, 1.15)
04	1.09(0.98, 1.20)	1.09(0.99, 1.20)	**1.11(1.02, 1.21)**	**1.15(1.03, 1.28)**	1.06(0.98, 1.15)	1.02(0.92, 1.15)
05	1.08(0.97, 1.20)	1.09(0.99, 1.21)	**1.09(1.00, 1.19)**	**1.16(1.04, 1.30)**	1.06(0.98, 1.16)	1.01(0.90, 1.13)
06	1.08(0.97, 1.20)	1.09(0.98, 1.21)	1.08(0.99, 1.19)	**1.17(1.04, 1.32)**	1.06(0.97, 1.16)	1.02(0.90, 1.15)
07	1.10(0.98, 1.23)	**1.12(1.00, 1.25)**	1.10(1.00, 1.20)	**1.19(1.05, 1.34)**	1.07(0.98, 1.17)	1.05(0.93, 1.19)

$P<0.05$ are highlighted in bold. The model covariates include long-term and seasonal trends, temperature, relative humidity, day of week, holiday and COVID-19.

Table S3 The results (OR with 95% *CI*) of daily anxiety visits associated with IQR increase in $PM_{2.5}$, PM_{10}, CO, SO_2, NO_2 and O_3 stratified by sex.

	Lag days	$PM_{2.5}$	PM_{10}	CO	NO_2	SO_2	O_3
Female	0	1.02(0.94, 1.12)	1.04(0.96, 1.13)	**1.15(1.06, 1.24)**	1.05(0.96, 1.14)	0.99(0.91, 1.07)	1.01(0.91, 1.13)
	1	**1.08(1.00, 1.17)**	1.06(0.99, 1.15)	**1.15(1.06, 1.24)**	1.04(0.96, 1.13)	1.02(0.95, 1.10)	1.02(0.93, 1.11)
	2	1.06(0.98, 1.14)	1.06(0.99, 1.14)	**1.11(1.03, 1.20)**	1.04(0.96, 1.12)	1.05(0.98, 1.13)	1.01(0.93, 1.09)
	3	1.05(0.97, 1.12)	1.05(0.98, 1.13)	1.05(0.98, 1.14)	1.07(0.99, 1.15)	1.04(0.97, 1.11)	1.01(0.93, 1.09)
	4	1.03(0.96, 1.11)	1.05(0.98, 1.12)	1.01(0.94, 1.10)	**1.07(1.00, 1.15)**	1.05(0.98, 1.12)	1.00(0.93, 1.08)
	5	1.03(0.96, 1.10)	1.03(0.96, 1.11)	1.02(0.94, 1.10)	1.07(0.99, 1.14)	1.04(0.97, 1.11)	0.97(0.90, 1.05)
	6	1.05(0.97, 1.12)	1.05(0.98, 1.13)	1.00(0.93, 1.08)	1.05(0.98, 1.12)	1.03(0.96, 1.10)	1.03(0.96, 1.11)
	7	**1.07(1.00, 1.15)**	**1.09(1.02, 1.17)**	1.05(0.98, 1.14)	1.07(1.00, 1.14)	**1.08(1.01, 1.15)**	**1.11(1.03, 1.19)**
	01	1.08(0.98, 1.18)	1.07(0.98, 1.17)	**1.18(1.09, 1.29)**	1.07(0.96, 1.18)	1.01(0.92, 1.10)	1.02(0.91, 1.15)
	02	1.09(0.99, 1.21)	1.09(0.99, 1.20)	**1.19(1.09, 1.30)**	1.08(0.97, 1.21)	1.03(0.94, 1.13)	1.02(0.90, 1.15)
	03	1.10(0.99, 1.23)	1.10(0.99, 1.22)	**1.17(1.07, 1.29)**	1.11(0.99, 1.25)	1.04(0.95, 1.14)	1.02(0.90, 1.16)
	04	1.11(0.99, 1.24)	1.11(1.00, 1.24)	**1.15(1.05, 1.27)**	**1.14(1.01, 1.29)**	1.05(0.96, 1.16)	1.02(0.89, 1.16)
	05	1.11(0.98, 1.25)	1.12(0.99, 1.25)	**1.14(1.03, 1.27)**	**1.17(1.02, 1.33)**	1.06(0.96, 1.16)	1.00(0.87, 1.14)
	06	1.12(0.99, 1.28)	1.13(1.00, 1.27)	**1.13(1.02, 1.26)**	**1.18(1.03, 1.35)**	1.06(0.96, 1.17)	1.01(0.88, 1.16)
	07	**1.15(1.01, 1.31)**	**1.16(1.02, 1.31)**	**1.14(1.02, 1.27)**	**1.20(1.04, 1.38)**	1.07(0.97, 1.19)	1.06(0.91, 1.22)

Male	0	1.00(0.88, 1.14)	1.03(0.91, 1.18)	0.99(0.88, 1.12)	0.95(0.83, 1.09)	1.07(0.94, 1.21)	0.91(0.78, 1.08)
	1	1.07(0.96, 1.21)	1.07(0.95, 1.19)	1.03(0.92, 1.16)	0.99(0.88, 1.12)	1.05(0.94, 1.17)	1.06(0.92, 1.21)
	2	1.03(0.93, 1.15)	1.03(0.93, 1.15)	1.03(0.92, 1.16)	1.09(0.97, 1.22)	1.02(0.92, 1.13)	1.07(0.95, 1.21)
	3	1.02(0.91, 1.13)	1.02(0.92, 1.14)	1.02(0.91, 1.14)	**1.16(1.04, 1.29)**	1.07(0.97, 1.19)	1.04(0.92, 1.16)
	4	0.99(0.89, 1.10)	1.00(0.90, 1.11)	0.98(0.87, 1.10)	1.08(0.98, 1.20)	1.06(0.96, 1.17)	0.98(0.88, 1.10)
	5	0.96(0.86, 1.06)	0.99(0.89, 1.10)	0.91(0.82, 1.02)	1.02(0.92, 1.13)	1.01(0.92, 1.12)	1.01(0.90, 1.13)
	6	0.93(0.84, 1.04)	0.96(0.87, 1.07)	0.97(0.87, 1.09)	1.02(0.92, 1.13)	1.00(0.90, 1.10)	0.99(0.89, 1.11)
	7	1.02(0.92, 1.13)	1.03(0.93, 1.13)	1.08(0.96, 1.20)	1.04(0.94, 1.15)	1.02(0.92, 1.12)	1.02(0.91, 1.14)
	01	1.05(0.92, 1.21)	1.07(0.93, 1.22)	1.02(0.89, 1.15)	0.96(0.83, 1.12)	1.07(0.94, 1.22)	0.99(0.83, 1.19)
	02	1.06(0.91, 1.23)	1.07(0.92, 1.24)	1.03(0.90, 1.17)	1.03(0.87, 1.22)	1.06(0.93, 1.22)	1.05(0.87, 1.26)
	03	1.06(0.90, 1.24)	1.07(0.91, 1.25)	1.03(0.89, 1.18)	1.13(0.95, 1.35)	1.08(0.94, 1.24)	1.06(0.88, 1.28)
	04	1.04(0.88, 1.23)	1.05(0.90, 1.24)	1.02(0.88, 1.17)	1.17(0.97, 1.40)	1.09(0.95, 1.26)	1.04(0.86, 1.26)
	05	1.01(0.85, 1.21)	1.04(0.87, 1.24)	0.99(0.85, 1.15)	1.16(0.95, 1.41)	1.08(0.93, 1.25)	1.04(0.85, 1.27)
	06	0.98(0.81, 1.18)	1.01(0.85, 1.22)	0.98(0.84, 1.15)	1.15(0.94, 1.41)	1.07(0.92, 1.24)	1.03(0.84, 1.26)
	07	0.99(0.82, 1.20)	1.03(0.85, 1.24)	1.00(0.85, 1.17)	1.17(0.95, 1.44)	1.07(0.92, 1.25)	1.04(0.84, 1.29)

$P < 0.05$ are highlighted in bold

Table S4 Comparison of the effects of air pollutants on daily anxiety visits in different gender groups

Lag days	Male VS Female					
	$PM_{2.5}$	PM_{10}	CO	NO_2	SO_2	O_3
0	-0.001(-0.011,0.008)	0.000(-0.006,0.006)	**-0.730(-1.436,-0.023)**	-0.009(-0.024,0.006)	0.039(-0.035,0.114)	-0.002(-0.005,0.001)
1	-0.001(-0.009,0.008)	0.000(-0.005,0.006)	-0.538(-1.233,0.158)	-0.005(-0.019,0.009)	0.014(-0.052,0.081)	0.001(-0.002,0.003)
2	-0.001(-0.010,0.007)	-0.001(-0.006,0.004)	-0.378(-1.064,0.308)	0.005(-0.008,0.017)	-0.014(-0.077,0.048)	0.001(-0.001,0.003)
3	-0.002(-0.010,0.006)	-0.001(-0.006,0.004)	-0.181(-0.866,0.503)	0.008(-0.004,0.020)	0.016(-0.045,0.076)	0.000(-0.002,0.003)
4	-0.003(-0.011,0.005)	-0.002(-0.007,0.003)	-0.174(-0.858,0.511)	0.001(-0.011,0.013)	0.005(-0.055,0.065)	0.000(-0.002,0.002)
5	-0.005(-0.013,0.003)	-0.002(-0.007,0.003)	-0.537(-1.220,0.147)	-0.004(-0.016,0.008)	-0.013(-0.072,0.047)	0.001(-0.001,0.003)
6	-0.007(-0.015,0.001)	-0.004(-0.008,0.001)	-0.172(-0.853,0.510)	-0.002(-0.014,0.009)	-0.017(-0.076,0.042)	-0.001(-0.003,0.001)
7	-0.003(-0.011,0.005)	-0.002(-0.007,0.002)	0.115(-0.558,0.789)	-0.003(-0.014,0.009)	-0.028(-0.087,0.032)	-0.001(-0.003,0.001)
01	-0.001(-0.012,0.009)	0.000(-0.007,0.006)	-0.756(-1.520,0.008)	-0.010(-0.027,0.008)	0.032(-0.047,0.111)	0.000(-0.004,0.003)
02	-0.002(-0.013,0.009)	-0.001(-0.008,0.006)	-0.736(-1.540,0.067)	-0.004(-0.023,0.015)	0.015(-0.067,0.096)	0.000(-0.003,0.004)
03	-0.003(-0.015,0.009)	-0.001(-0.009,0.006)	-0.672(-1.509,0.164)	0.002(-0.018,0.022)	0.019(-0.064,0.102)	0.001(-0.003,0.004)
04	-0.004(-0.016,0.009)	-0.002(-0.010,0.006)	-0.642(-1.513,0.229)	0.002(-0.019,0.023)	0.018(-0.067,0.103)	0.000(-0.003,0.004)
05	-0.006(-0.019,0.008)	-0.003(-0.011,0.005)	-0.735(-1.639,0.169)	-0.001(-0.023,0.022)	0.011(-0.076,0.098)	0.001(-0.003,0.004)
06	-0.009(-0.023,0.005)	-0.004(-0.013,0.004)	-0.722(-1.657,0.213)	-0.002(-0.025,0.021)	0.004(-0.085,0.093)	0.000(-0.004,0.004)
07	-0.009(-0.024,0.005)	-0.005(-0.014,0.004)	-0.654(-1.619,0.310)	-0.002(-0.026,0.022)	-0.001(-0.093,0.090)	0.000(-0.004,0.004)

$P < 0.05$ are highlighted in bold, indicating statistical significance in the differences.

Table S5 The results (OR with 95% *CI*) of daily anxiety visits associated with IQR increase in $PM_{2.5}$, PM_{10}, CO, SO_2, NO_2 and O_3 stratified by age

Age	Lag days	$PM_{2.5}$	PM_{10}	CO	SO_2	NO_2	O_3
＜45	0	1.07(0.94, 1.23)	1.10(0.97, 1.26)	0.99(0.88, 1.12)	1.00(0.88, 1.14)	1.01(0.88, 1.16)	1.03(0.87, 1.21)
	1	1.04(0.92, 1.17)	1.04(0.92, 1.17)	0.99(0.87, 1.12)	1.03(0.92, 1.16)	0.97(0.85, 1.11)	1.09(0.95, 1.25)
	2	1.02(0.91, 1.14)	1.01(0.90, 1.13)	1.04(0.93, 1.18)	0.99(0.89, 1.10)	1.06(0.95, 1.20)	1.03(0.90, 1.16)
	3	1.05(0.94, 1.17)	1.03(0.93, 1.15)	1.02(0.90, 1.15)	1.04(0.94, 1.15)	1.12(1.00, 1.25)	1.05(0.93, 1.18)
	4	1.05(0.94, 1.17)	1.04(0.93, 1.16)	1.02(0.91, 1.15)	1.05(0.95, 1.16)	1.10(0.99, 1.23)	1.02(0.91, 1.15)
	5	1.02(0.92, 1.14)	1.01(0.91, 1.13)	0.98(0.87, 1.11)	1.00(0.90, 1.10)	1.05(0.94, 1.18)	0.97(0.86, 1.09)
	6	0.99(0.89, 1.11)	0.99(0.89, 1.10)	1.04(0.93, 1.17)	0.97(0.87, 1.07)	0.98(0.88, 1.10)	1.04(0.93, 1.17)
	7	1.06(0.95, 1.18)	1.05(0.95, 1.17)	**1.14(1.01, 1.28)**	1.01(0.91, 1.12)	1.02(0.92, 1.14)	1.11(0.99, 1.25)
	01	1.07(0.93, 1.24)	1.09(0.95, 1.25)	0.99(0.86, 1.13)	1.02(0.90, 1.17)	0.99(0.84, 1.16)	1.09(0.91, 1.31)
	02	1.06(0.91, 1.24)	1.07(0.92, 1.24)	1.01(0.88, 1.16)	1.01(0.88, 1.16)	1.03(0.87, 1.23)	1.08(0.90, 1.31)
	03	1.08(0.91, 1.27)	1.07(0.92, 1.26)	1.01(0.88, 1.18)	1.03(0.89, 1.18)	1.10(0.92, 1.33)	1.10(0.91, 1.34)
	04	1.09(0.92, 1.30)	1.08(0.91, 1.28)	1.02(0.88, 1.19)	1.04(0.90, 1.20)	1.15(0.95, 1.40)	1.10(0.90, 1.34)
	05	1.10(0.91, 1.32)	1.08(0.91, 1.30)	1.01(0.86, 1.19)	1.03(0.89, 1.20)	1.18(0.96, 1.45)	1.07(0.87, 1.31)
	06	1.09(0.89, 1.32)	1.07(0.88, 1.29)	1.02(0.87, 1.20)	1.02(0.87, 1.19)	1.14(0.92, 1.42)	1.08(0.87, 1.34)
	07	1.11(0.91, 1.37)	1.09(0.90, 1.32)	1.06(0.89, 1.25)	1.02(0.87, 1.19)	1.15(0.92, 1.44)	1.13(0.91, 1.42)

45-64	0	0.95(0.85, 1.06)	0.96(0.86, 1.07)	**1.16(1.05, 1.27)**	0.99(0.89, 1.10)	1.01(0.91, 1.13)	0.95(0.83, 1.08)
	1	1.10(1.00, 1.21)	1.08(0.99, 1.19)	**1.20(1.09, 1.32)**	1.04(0.95, 1.14)	1.09(0.98, 1.20)	0.98(0.88, 1.09)
	2	**1.10(1.01, 1.20)**	**1.12(1.03, 1.22)**	**1.10(1.00, 1.21)**	**1.09(1.00, 1.19)**	**1.10(1.00, 1.20)**	1.06(0.96, 1.17)
	3	1.05(0.96, 1.15)	1.08(0.99, 1.17)	1.06(0.96, 1.16)	1.06(0.97, 1.15)	**1.10(1.01, 1.20)**	1.02(0.92, 1.12)
	4	1.04(0.96, 1.14)	1.08(0.99, 1.17)	0.99(0.90, 1.08)	**1.09(1.00, 1.18)**	**1.10(1.01, 1.20)**	1.03(0.94, 1.13)
	5	1.01(0.92, 1.10)	1.03(0.95, 1.12)	0.98(0.89, 1.07)	1.07(0.99, 1.16)	1.06(0.97, 1.15)	0.98(0.89, 1.07)
	6	1.02(0.94, 1.11)	1.05(0.97, 1.14)	0.96(0.87, 1.06)	1.06(0.98, 1.15)	1.07(0.98, 1.16)	1.00(0.91, 1.10)
	7	1.05(0.97, 1.15)	**1.08(1.00, 1.17)**	1.02(0.93, 1.12)	1.07(0.99, 1.16)	1.08(1.00, 1.18)	1.08(0.98, 1.18)
	01	1.04(0.93, 1.17)	1.04(0.93, 1.16)	**1.22(1.10, 1.35)**	1.03(0.92, 1.14)	1.07(0.95, 1.22)	0.95(0.82, 1.10)
	02	1.09(0.97, 1.24)	1.10(0.98, 1.24)	**1.21(1.08, 1.35)**	1.07(0.95, 1.19)	1.13(0.99, 1.30)	1.00(0.86, 1.17)
	03	1.11(0.97, 1.26)	1.13(0.99, 1.28)	**1.19(1.06, 1.34)**	1.08(0.96, 1.21)	**1.18(1.02, 1.37)**	1.01(0.87, 1.19)
	04	1.12(0.97, 1.29)	**1.15(1.01, 1.32)**	**1.16(1.03, 1.31)**	1.10(0.98, 1.24)	**1.23(1.05, 1.43)**	1.03(0.88, 1.21)
	05	1.11(0.95, 1.28)	1.15(0.99, 1.33)	**1.13(1.00, 1.29)**	1.11(0.98, 1.25)	**1.24(1.05, 1.45)**	1.01(0.86, 1.19)
	06	1.11(0.95, 1.29)	1.16(1.00, 1.35)	1.11(0.98, 1.27)	1.12(0.99, 1.26)	**1.26(1.06, 1.49)**	1.01(0.85, 1.20)
	07	1.12(0.96, 1.32)	**1.18(1.02, 1.38)**	1.11(0.97, 1.27)	1.13(0.99, 1.27)	**1.28(1.08, 1.52)**	1.04(0.88, 1.24)
⩾65	0	1.10(0.94, 1.27)	1.12(0.97, 1.30)	1.13(0.99, 1.30)	1.06(0.91, 1.22)	1.06(0.91, 1.23)	1.01(0.83, 1.22)
	1	1.11(0.97, 1.27)	1.06(0.93, 1.21)	1.11(0.97, 1.27)	1.01(0.88, 1.15)	0.98(0.85, 1.13)	1.07(0.91, 1.25)
	2	0.98(0.86, 1.12)	0.96(0.85, 1.09)	1.12(0.98, 1.28)	1.01(0.90, 1.15)	0.95(0.83, 1.08)	0.95(0.82, 1.10)

3	0.99(0.87, 1.13)	0.99(0.87, 1.11)	1.05(0.91, 1.20)	1.04(0.93, 1.18)	1.05(0.93, 1.18)	0.96(0.84, 1.11)
4	0.91(0.80, 1.04)	0.92(0.81, 1.05)	1.01(0.88, 1.16)	0.97(0.86, 1.09)	0.99(0.88, 1.13)	0.88(0.77, 1.01)
5	0.98(0.87, 1.12)	1.00(0.89, 1.13)	1.00(0.88, 1.15)	0.98(0.87, 1.10)	1.04(0.92, 1.17)	1.02(0.89, 1.16)
6	1.01(0.89, 1.14)	1.02(0.91, 1.15)	1.01(0.88, 1.15)	0.99(0.88, 1.12)	1.05(0.93, 1.18)	1.02(0.89, 1.16)
7	1.06(0.94, 1.20)	1.08(0.96, 1.21)	1.04(0.91, 1.19)	1.11(0.99, 1.25)	1.06(0.94, 1.19)	1.03(0.90, 1.18)
01	1.14(0.97, 1.34)	1.12(0.95, 1.31)	1.15(0.99, 1.33)	1.04(0.89, 1.21)	1.02(0.86, 1.21)	1.06(0.86, 1.31)
02	1.09(0.91, 1.30)	1.06(0.89, 1.26)	1.16(0.99, 1.36)	1.03(0.88, 1.21)	0.97(0.80, 1.18)	1.01(0.81, 1.25)
03	1.07(0.89, 1.29)	1.04(0.87, 1.25)	1.15(0.98, 1.36)	1.04(0.89, 1.23)	1.00(0.82, 1.23)	0.98(0.78, 1.23)
04	1.01(0.83, 1.24)	0.99(0.82, 1.20)	1.13(0.95, 1.35)	1.02(0.87, 1.21)	1.00(0.81, 1.23)	0.91(0.73, 1.15)
05	1.00(0.81, 1.24)	0.99(0.80, 1.21)	1.12(0.94, 1.34)	1.01(0.85, 1.20)	1.01(0.81, 1.27)	0.93(0.73, 1.18)
06	1.00(0.80, 1.25)	0.99(0.80, 1.23)	1.11(0.93, 1.34)	1.00(0.84, 1.20)	1.03(0.82, 1.30)	0.94(0.73, 1.20)
07	1.03(0.82, 1.29)	1.02(0.82, 1.27)	1.12(0.92, 1.36)	1.03(0.86, 1.24)	1.06(0.83, 1.34)	0.96(0.74, 1.23)

$P < 0.05$ are highlighted in bold, indicating statistical significance in the differences.

Table S6 Comparison of the effects of air pollutants on daily anxiety visits between young-aged group and middle-aged group

Lag days	＜45 VS 45-64					
	$PM_{2.5}$	PM_{10}	CO	NO_2	SO_2	O_3
0	0.008(-0.003,0.018)	0.008(-0.003,0.018)	-0.775(-1.555,0.006)	0.000(-0.017,0.017)	-0.775(-1.555,0.006)	0.001(-0.002,0.005)
1	-0.004(-0.013,0.006)	-0.004(-0.013,0.006)	**-0.969(-1.741,-0.197)**	-0.011(-0.026,0.005)	**-0.969(-1.741,-0.197)**	0.002(-0.001,0.004)
2	-0.005(-0.014,0.004)	-0.005(-0.014,0.004)	-0.271(-1.033,0.490)	-0.003(-0.017,0.011)	-0.271(-1.033,0.490)	-0.001(-0.003,0.002)
3	0.000(-0.009,0.009)	0.000(-0.009,0.009)	-0.180(-0.940,0.580)	0.001(-0.012,0.015)	-0.180(-0.940,0.580)	0.001(-0.002,0.003)
4	0.000(-0.008,0.009)	0.000(-0.008,0.009)	0.187(-0.571,0.944)	0.000(-0.013,0.014)	0.187(-0.571,0.944)	0.000(-0.003,0.002)
5	0.001(-0.008,0.010)	0.001(-0.008,0.010)	0.027(-0.729,0.782)	-0.001(-0.014,0.013)	0.027(-0.729,0.782)	0.000(-0.002,0.002)
6	-0.002(-0.011,0.007)	-0.002(-0.011,0.007)	0.398(-0.357,1.152)	-0.008(-0.021,0.005)	0.398(-0.357,1.152)	0.001(-0.002,0.003)
7	0.000(-0.008,0.009)	0.000(-0.008,0.009)	0.523(-0.224,1.270)	-0.005(-0.018,0.008)	0.523(-0.224,1.270)	0.001(-0.002,0.003)
01	0.002(-0.010,0.013)	0.002(-0.010,0.013)	**-1.041(-1.889,-0.194)**	-0.008(-0.028,0.011)	**-1.041(-1.889,-0.194)**	0.002(-0.001,0.006)
02	-0.002(-0.014,0.011)	-0.002(-0.014,0.011)	**-0.897(-1.789,-0.005)**	-0.009(-0.030,0.012)	**-0.897(-1.789,-0.005)**	0.001(-0.003,0.005)
03	-0.002(-0.015,0.011)	-0.002(-0.015,0.011)	-0.802(-1.733,0.128)	-0.007(-0.029,0.016)	-0.802(-1.733,0.128)	0.001(-0.003,0.005)
04	-0.001(-0.015,0.013)	-0.001(-0.015,0.013)	-0.638(-1.607,0.330)	-0.006(-0.029,0.018)	-0.638(-1.607,0.330)	0.001(-0.003,0.005)
05	0.000(-0.015,0.014)	0.000(-0.015,0.014)	-0.571(-1.577,0.434)	-0.005(-0.030,0.020)	-0.571(-1.577,0.434)	0.001(-0.003,0.005)
06	-0.001(-0.017,0.014)	-0.001(-0.017,0.014)	-0.417(-1.456,0.623)	-0.009(-0.035,0.017)	-0.417(-1.456,0.623)	0.001(-0.003,0.005)
07	-0.001(-0.017,0.016)	-0.001(-0.017,0.016)	-0.261(-1.335,0.812)	-0.010(-0.037,0.017)	-0.261(-1.335,0.812)	0.001(-0.003,0.006)

$P < 0.05$ are highlighted in bold, indicating statistical significance in the differences.

Table S7 Comparison of the effects of air pollutants on daily anxiety visits between young-aged group and old-aged group

Lag days	<45 VS ≥65					
	$PM_{2.5}$	PM_{10}	CO	NO_2	SO_2	O_3
0	-0.001(-0.014,0.011)	-0.001(-0.009,0.007)	-0.658(-1.588,0.272)	-0.004(-0.024,0.015)	-0.658(-1.588,0.272)	0.000(-0.004,0.004)
1	-0.004(-0.015,0.007)	-0.001(-0.008,0.006)	-0.579(-1.498,0.339)	0.000(-0.019,0.018)	-0.579(-1.498,0.339)	0.000(-0.003,0.004)
2	0.002(-0.009,0.013)	0.002(-0.005,0.009)	-0.336(-1.236,0.564)	0.011(-0.005,0.028)	-0.336(-1.236,0.564)	0.001(-0.002,0.004)
3	0.003(-0.007,0.014)	0.002(-0.005,0.009)	-0.144(-1.047,0.759)	0.006(-0.010,0.022)	-0.144(-1.047,0.759)	0.001(-0.001,0.004)
4	0.009(-0.002,0.019)	0.005(-0.002,0.011)	0.048(-0.854,0.949)	0.010(-0.006,0.026)	0.048(-0.854,0.949)	0.002(-0.001,0.005)
5	0.002(-0.008,0.013)	0.001(-0.006,0.007)	-0.109(-1.006,0.788)	0.001(-0.014,0.017)	-0.109(-1.006,0.788)	-0.001(-0.004,0.002)
6	-0.001(-0.012,0.009)	-0.001(-0.008,0.005)	0.154(-0.739,1.048)	-0.006(-0.022,0.009)	0.154(-0.739,1.048)	0.000(-0.002,0.003)
7	0.000(-0.010,0.010)	-0.001(-0.007,0.005)	0.427(-0.461,1.314)	-0.003(-0.019,0.012)	0.427(-0.461,1.314)	0.001(-0.002,0.004)
01	-0.004(-0.017,0.010)	-0.001(-0.010,0.007)	-0.739(-1.748,0.270)	-0.003(-0.026,0.019)	-0.739(-1.748,0.270)	0.000(-0.004,0.005)
02	-0.002(-0.016,0.013)	0.000(-0.009,0.009)	-0.702(-1.762,0.359)	0.006(-0.019,0.030)	-0.702(-1.762,0.359)	0.001(-0.003,0.006)
03	0.000(-0.015,0.016)	0.001(-0.008,0.011)	-0.628(-1.735,0.479)	0.009(-0.017,0.035)	-0.628(-1.735,0.479)	0.002(-0.003,0.006)
04	0.005(-0.012,0.021)	0.003(-0.007,0.014)	-0.531(-1.683,0.620)	0.014(-0.014,0.041)	-0.531(-1.683,0.620)	0.003(-0.002,0.007)
05	0.006(-0.012,0.023)	0.004(-0.007,0.015)	-0.513(-1.710,0.683)	0.014(-0.015,0.043)	-0.513(-1.710,0.683)	0.002(-0.003,0.007)
06	0.005(-0.014,0.023)	0.003(-0.008,0.014)	-0.428(-1.666,0.810)	0.010(-0.020,0.040)	-0.428(-1.666,0.810)	0.002(-0.003,0.007)
07	0.005(-0.014,0.024)	0.003(-0.009,0.014)	-0.297(-1.574,0.980)	0.008(-0.023,0.039)	-0.297(-1.574,0.980)	0.003(-0.003,0.008)

$P < 0.05$ are highlighted in bold, indicating statistical significance in the differences.

Table S8 Comparison of the effects of air pollutants on daily anxiety visits between middle-aged group and old-aged group

Lag days	45-64 VS ≥65					
	$PM_{2.5}$	PM_{10}	CO	NO_2	SO_2	O_3
0	-0.009(-0.020,0.003)	-0.006(-0.013,0.001)	0.117(-0.724,0.957)	-0.004(-0.022,0.014)	0.117(-0.724,0.957)	-0.001(-0.005,0.003)
1	-0.001(-0.011,0.010)	0.001(-0.006,0.007)	0.390(-0.441,1.220)	0.010(-0.006,0.027)	0.390(-0.441,1.220)	-0.001(-0.004,0.002)
2	0.007(-0.003,0.017)	**0.006(0.000,0.012)**	-0.065(-0.885,0.756)	0.014(-0.001,0.030)	-0.065(-0.885,0.756)	0.002(-0.001,0.004)
3	0.004(-0.006,0.013)	0.004(-0.002,0.010)	0.036(-0.791,0.863)	0.005(-0.010,0.019)	0.036(-0.791,0.863)	0.001(-0.002,0.003)
4	0.008(-0.001,0.018)	**0.006(0.000,0.012)**	-0.139(-0.969,0.691)	0.010(-0.005,0.024)	-0.139(-0.969,0.691)	0.002(0.000,0.005)
5	0.001(-0.008,0.011)	0.001(-0.005,0.007)	-0.136(-0.956,0.685)	0.002(-0.012,0.016)	-0.136(-0.956,0.685)	-0.001(-0.003,0.002)
6	0.001(-0.009,0.010)	0.001(-0.005,0.007)	-0.243(-1.066,0.579)	0.002(-0.012,0.016)	-0.243(-1.066,0.579)	0.000(-0.003,0.002)
7	0.000(-0.010,0.009)	0.000(-0.005,0.006)	-0.096(-0.914,0.721)	0.002(-0.012,0.016)	-0.096(-0.914,0.721)	0.001(-0.002,0.003)
01	-0.006(-0.018,0.007)	-0.003(-0.011,0.005)	0.302(-0.612,1.216)	0.005(-0.015,0.026)	0.302(-0.612,1.216)	-0.002(-0.006,0.002)
02	0.000(-0.013,0.014)	0.002(-0.007,0.010)	0.195(-0.767,1.158)	0.015(-0.008,0.037)	0.195(-0.767,1.158)	0.000(-0.004,0.004)
03	0.002(-0.012,0.016)	0.003(-0.006,0.012)	0.174(-0.833,1.182)	0.016(-0.008,0.039)	0.174(-0.833,1.182)	0.001(-0.004,0.005)
04	0.006(-0.009,0.021)	0.006(-0.003,0.015)	0.107(-0.945,1.159)	0.020(-0.005,0.045)	0.107(-0.945,1.159)	0.002(-0.002,0.006)
05	0.006(-0.010,0.022)	0.006(-0.004,0.016)	0.058(-1.036,1.153)	0.019(-0.007,0.045)	0.058(-1.036,1.153)	0.001(-0.003,0.006)
06	0.006(-0.011,0.023)	0.006(-0.004,0.017)	-0.012(-1.146,1.123)	0.019(-0.008,0.046)	-0.012(-1.146,1.123)	0.001(-0.004,0.006)
07	0.006(-0.012,0.023)	0.006(-0.005,0.017)	-0.036(-1.206,1.135)	0.019(-0.009,0.047)	-0.036(-1.206,1.135)	0.001(-0.003,0.006)

$P < 0.05$ are highlighted in bold, indicating statistical significance in the differences.

Table S9 The results (OR with 95% *CI*) of daily anxiety visits associated with IQR increase in $PM_{2.5}$, PM_{10}, SO_2, NO_2 CO and O_3 for different degrees of freedom

df for time (per year)	$PM_{2.5}$	PM_{10}	CO	NO_2	SO_2	O_3
5	**1.08(1.01, 1.16)**	**1.12(1.00, 1.25)**	**1.14(1.05, 1.23)**	**1.19(1.05, 1.34)**	**1.06(1.00, 1.12)**	**1.08(1.01, 1.15)**
3	1.06(0.99, 1.14)	1.10(0.98, 1.22)	**1.18(1.09, 1.27)**	**1.16(1.03, 1.31)**	1.05(1.00, 1.11)	**1.09(1.02, 1.17)**
4	**1.07(1.00, 1.15)**	1.10(0.99, 1.23)	**1.16(1.08, 1.26)**	**1.17(1.04, 1.33)**	**1.09(1.03, 1.15)**	**1.09(1.03, 1.17)**
6	1.07(1.00, 1.14)	1.07(0.96, 1.20)	**1.11(1.02, 1.20)**	**1.17(1.03, 1.32)**	1.04(0.98, 1.11)	1.06(0.99, 1.13)
7	1.07(1.00, 1.14)	1.08(0.96, 1.20)	**1.10(1.01, 1.19)**	**1.14(1.01, 1.29)**	1.05(0.99, 1.12)	1.06(1.00, 1.13)

$P < 0.05$ are highlighted in bold; $PM_{2.5}$ at lag1, PM_{10} at lag07, CO at lag02, NO_2 at lag07, SO_2 at lag7, O_3 at lag7

Table S10 The results (OR with 95% *CI*) of daily anxiety visits associated with IQR increase in $PM_{2.5}$, PM_{10}, CO, SO_2, NO_2 and O_3 in two-pollutant models

	Two pollutants models	OR (95%*CI*)
**$PM_{2.5}$**	-	**1.08(1.01, 1.16)**
	+CO	1.02(0.96, 1.09)
	+NO_2	**1.10(1.01, 1.20)**
	+SO_2	**1.08(1.00, 1.17)**
	+O_3	**1.10(1.02, 1.20)**
**PM_{10}**	-	**1.12(1.00, 1.25)**
	+CO	1.10(0.98, 1.23)
	+NO_2	1.02(0.89, 1.18)
	+SO_2	1.10(0.96, 1.25)
	+O_3	**1.21(1.02, 1.43)**
**CO**	-	**1.14(1.05, 1.23)**
	+$PM_{2.5}$	**1.13(1.04, 1.23)**
	+PM_{10}	**1.13(1.04, 1.22)**
	+NO_2	**1.13(1.05, 1.23)**
	+SO_2	**1.16(1.07, 1.26)**
	+O_3	**1.14(1.05, 1.23)**
**NO_2**	-	**1.19(1.05, 1.34)**
	+$PM_{2.5}$	**1.19(1.03, 1.39)**
	+PM_{10}	**1.17(1.00, 1.37)**
	+CO	**1.17(1.03, 1.32)**
	+SO_2	**1.17(1.03, 1.34)**
	+O_3	**1.21(1.06, 1.38)**
**SO_2**	-	**1.06(1.00, 1.12)**
	+$PM_{2.5}$	1.04(0.97, 1.11)

	$+PM_{10}$	1.02(0.95, 1.10)
	$+NO_2$	1.04(0.98, 1.11)
	+CO	**1.06(1.00, 1.13)**
	$+O_3$	1.03(0.96, 1.10)
**O_3**	-	**1.08(1.01, 1.15)**
	$+PM_{2.5}$	1.07(0.98, 1.17)
	$+PM_{10}$	1.04(0.95, 1.14)
	$+NO_2$	1.06(0.99, 1.14)
	+CO	**1.08(1.01, 1.15)**
	$+ SO_2$	1.06(0.98, 1.14)

P < 0.05 are highlighted in bold;

Table S11 The results (OR with 95% CI) of daily anxiety visits associated with IQR increase in $PM_{2.5}$, PM_{10}, CO, SO_2, NO_2 and O_3 for different lag structures, restricting analysis before January 8, 2023.

Lag days	$PM_{2.5}$	PM_{10}	CO	NO_2	SO_2	O_3
0	1.01(0.93, 1.10)	1.03(0.96, 1.11)	**1.10(1.03, 1.19)**	1.02(0.94, 1.10)	1.02(0.90, 1.15)	0.95(0.86, 1.05)
1	**1.09(1.02, 1.17)**	1.07(1.00, 1.14)	**1.11(1.03, 1.19)**	1.03(0.96, 1.11)	1.06(0.95, 1.19)	1.00(0.93, 1.09)
2	1.04(0.97, 1.11)	1.03(0.97, 1.10)	1.07(1.00, 1.15)	1.04(0.97, 1.11)	1.07(0.97, 1.19)	1.00(0.92, 1.08)
3	1.02(0.95, 1.09)	1.02(0.96, 1.08)	1.02(0.95, 1.10)	**1.07(1.01, 1.14)**	1.07(0.97, 1.18)	0.99(0.92, 1.06)
4	1.00(0.94, 1.07)	1.01(0.95, 1.07)	0.99(0.92, 1.06)	1.06(0.99, 1.13)	1.06(0.97, 1.17)	0.96(0.90, 1.03)
5	1.00(0.94, 1.07)	1.01(0.95, 1.07)	0.97(0.91, 1.04)	1.04(0.98, 1.10)	1.05(0.95, 1.15)	0.96(0.89, 1.03)
6	1.00(0.94, 1.07)	1.01(0.95, 1.07)	0.98(0.91, 1.05)	1.02(0.96, 1.08)	1.02(0.93, 1.12)	1.00(0.93, 1.07)
7	1.06(1.00, 1.13)	**1.07(1.01, 1.13)**	1.05(0.98, 1.12)	1.05(0.99, 1.12)	**1.10(1.00, 1.21)**	1.07(1.00, 1.14)
01	1.08(0.98, 1.17)	1.07(0.98, 1.16)	**1.13(1.04, 1.22)**	1.04(0.94, 1.13)	1.05(0.92, 1.20)	0.97(0.87, 1.08)
02	1.08(0.98, 1.19)	1.07(0.98, 1.17)	**1.13(1.04, 1.22)**	1.06(0.96, 1.17)	1.08(0.94, 1.24)	0.98(0.87, 1.09)
03	1.08(0.98, 1.19)	1.07(0.97, 1.17)	**1.11(1.02, 1.21)**	1.10(0.99, 1.22)	1.09(0.95, 1.26)	0.97(0.87, 1.10)
04	1.07(0.96, 1.19)	1.06(0.96, 1.17)	1.09(1.00, 1.19)	**1.12(1.00, 1.25)**	1.10(0.95, 1.27)	0.96(0.85, 1.08)
05	1.06(0.94, 1.19)	1.06(0.95, 1.17)	1.07(0.98, 1.18)	**1.13(1.00, 1.27)**	1.10(0.94, 1.28)	0.94(0.83, 1.06)
06	1.05(0.93, 1.19)	1.05(0.94, 1.17)	1.06(0.96, 1.17)	1.13(1.00, 1.27)	1.09(0.93, 1.27)	0.94(0.82, 1.07)
07	1.08(0.95, 1.22)	1.08(0.97, 1.21)	1.07(0.97, 1.19)	**1.14(1.01, 1.30)**	1.12(0.95, 1.32)	0.97(0.85, 1.12)

P<0.05 are highlighted in bold.
